# Dynamic Navigation-Assisted Coronectomy of a Deeply Impacted Mandibular Third Molar: A 5-Year Case Follow-Up

**DOI:** 10.1155/crid/7010729

**Published:** 2025-11-14

**Authors:** Gerardo Pellegrino, Subhi Tayeb, Elisabetta Vignudelli, Claudia Angelino, Carlo Barausse, Pietro Felice

**Affiliations:** Unit of Oral Surgery, Department of Biomedical and Neuromotor Sciences, University of Bologna, Bologna, Italy

**Keywords:** case report, computer-assisted surgery, coronectomy, dynamic navigation, impacted tooth, inferior alveolar nerve, long-term follow-up, oral surgery, third molar

## Abstract

Coronectomy is a conservative surgical technique used to manage deeply impacted mandibular third molars at high risk of inferior alveolar nerve injury. Precise execution is essential to avoid complications, particularly in cases with limited surgical access. Dynamic navigation (DN) systems may enhance accuracy and safety in such procedures. This report describes the 5-year follow-up of a DN-assisted coronectomy in a 42-year-old patient presenting with recurrent pericoronitis and a pericoronal lesion associated with a deeply impacted lower third molar. Preoperative planning was performed using cone beam computed tomography (CBCT), and DN was employed intraoperatively to guide surgical instrumentation in real time. The procedure was carried out according to a standardized protocol, including crown sectioning, root reduction, and primary closure. No intraoperative or early postoperative complications were observed. At 5-year follow-up, the patient was asymptomatic. Clinical examination showed complete mucosal healing and normal probing depths. Radiographic evaluation revealed retained roots without signs of pathology and bone formation distal to the second molar. This case may highlight the potential role of DN in improving surgical control during coronectomy in anatomically complex situations, contributing to a favorable long-term clinical and radiographic outcome.

## 1. Introduction

Coronectomy is a surgical approach for managing high neurological risk third molars, aimed at preventing or minimizing iatrogenic nerve injuries [1–4]. The surgical protocol involves the removal of only the crown of a high-risk third molar, leaving the residual roots in place [5–8]. Several short- and long-term studies have highlighted the positive outcomes achievable with this technique, such as a reduced risk of neurological damage and a low incidence of postoperative complications [9–13].

However, these favorable outcomes can only be expected if a correct and standardized surgical protocol is strictly followed. Indeed, standardization is essential to avoid technical errors, such as leaving residual enamel, which has been associated with an increased risk of complications [14, 15].

Barcellos et al. [15] reviewed 2062 coronectomies and reported a reoperation rate of 5.1% (range: 6 months to 10 years; mean: 10.4 months). Among the reasons for reintervention, 10% were attributed to irregular crown sectioning, which resulted in retained enamel that compromised the maintenance of the residual roots in situ. Furthermore, coronectomy is often indicated for third molars with close proximity to the mandibular canal, which are frequently deeply impacted and, consequently, difficult to access surgically, making adherence to the ideal protocol more challenging [16].

For these reasons, the use of modern technology, specifically dynamic navigation (DN), could provide benefits in oral and exodontic surgery [17].

DN employs a stereoscopic camera that detects the spatial relationship of reference markers placed on the patient and the surgical handpiece in real time. It also integrates these data with cone beam computed tomography (CBCT) images. The result is a real-time visualization of anatomical structures, offering continuous feedback consistent with the 3D preoperative plan [18, 19].

The application of DN in dentistry has expanded due to the increasing availability of CBCT and the development of dedicated software and user-friendly reference tools calibrated for dental use [20, 21].

In oral surgery, DN was initially used in isolated case reports [17] or in selected indications, such as supernumerary or ectopic tooth extraction [22]. A recent randomized controlled trial evaluated the safety and efficacy of DN in the extraction of deeply impacted horizontal mandibular third molars. The authors concluded that the precision and real-time guidance of DN support its use in complex extractions, potentially ushering in a new era in oral and maxillofacial surgery [23].

With regard to coronectomy, a recent pilot study by Zhang et al. [24] investigated the accuracy of DN, concluding that it may be helpful; however, no long-term data are currently available regarding complications related to residual roots when DN is used.

The present study reports the 5-year follow-up of a previously published case involving the coronectomy of a third molar in close proximity to the mandibular canal, performed with DN guidance [25].

To the authors' knowledge, this is the first report to evaluate both the procedural accuracy and the long-term complication rate associated with DN-assisted coronectomy.

## 2. Case Presentation

The patient was treated at the Unit of Oral Surgery, Department of Biomedical and Neuromotor Sciences (DIBINEM) at the University of Bologna, Italy. The objectives and procedures of the study were thoroughly explained to the patient, and informed consent was obtained. The study was conducted in adherence to the Declaration of Helsinki and was prepared in accordance with the CARE (CAse REport) guidelines to ensure completeness and transparency in clinical reporting. Ethical approval was obtained from the Ethics Committee of the Azienda Unità Sanitaria Locale Città di Bologna, Italy (Comitato Etico 12098, 2012).

**Figure 1:**
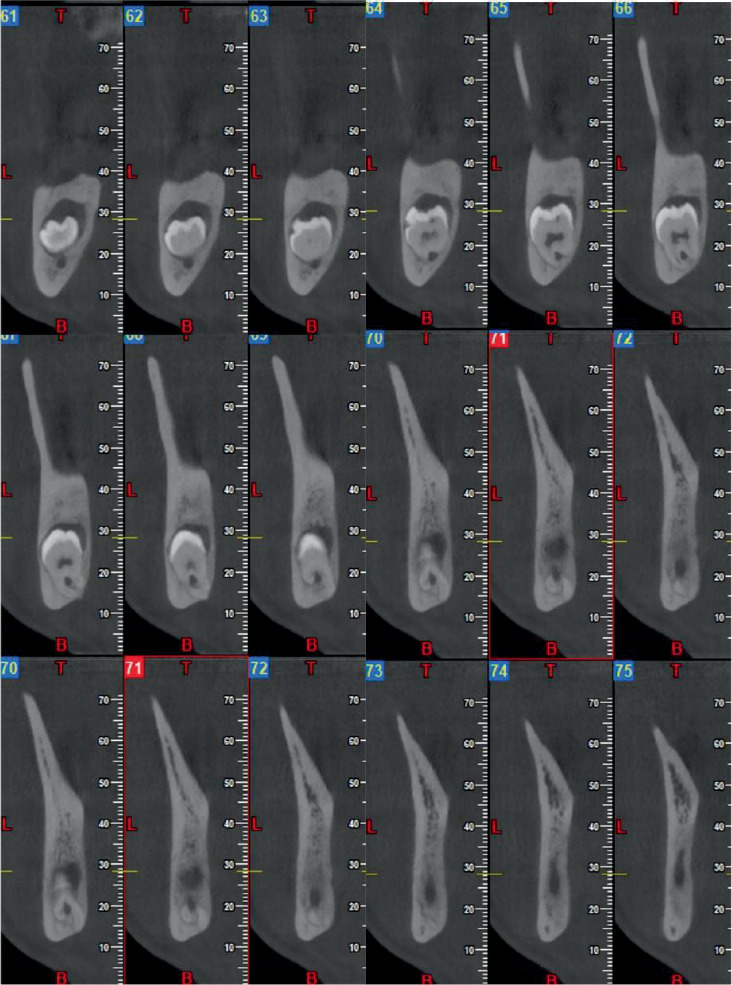
Preoperative cone beam computed tomography. The inferior alveolar nerve was trapped in the furcation roots of the lower third molar.

The patient presented with recurrent episodes of pericoronitis, previously managed with antibiotic therapy. Clinical and radiographic examination revealed a deeply impacted left mandibular third molar, associated with a pericoronal lesion and classified as high risk for neurological injury (Figure 1). The panoramic radiograph showed a vertically impacted left lower third molar classified as IC grade according to the Pell and Gregory scale.

Given the elevated neurological risk, the technical complexity of the case, and the patient's preference for a less invasive and more time-efficient solution, the decision was made to perform a coronectomy assisted by a DN system. The ImplaNav navigation system (BresMedical, Sydney, Australia) was used, in accordance with the previously published coronectomy protocol [25].

**Figure 2:**
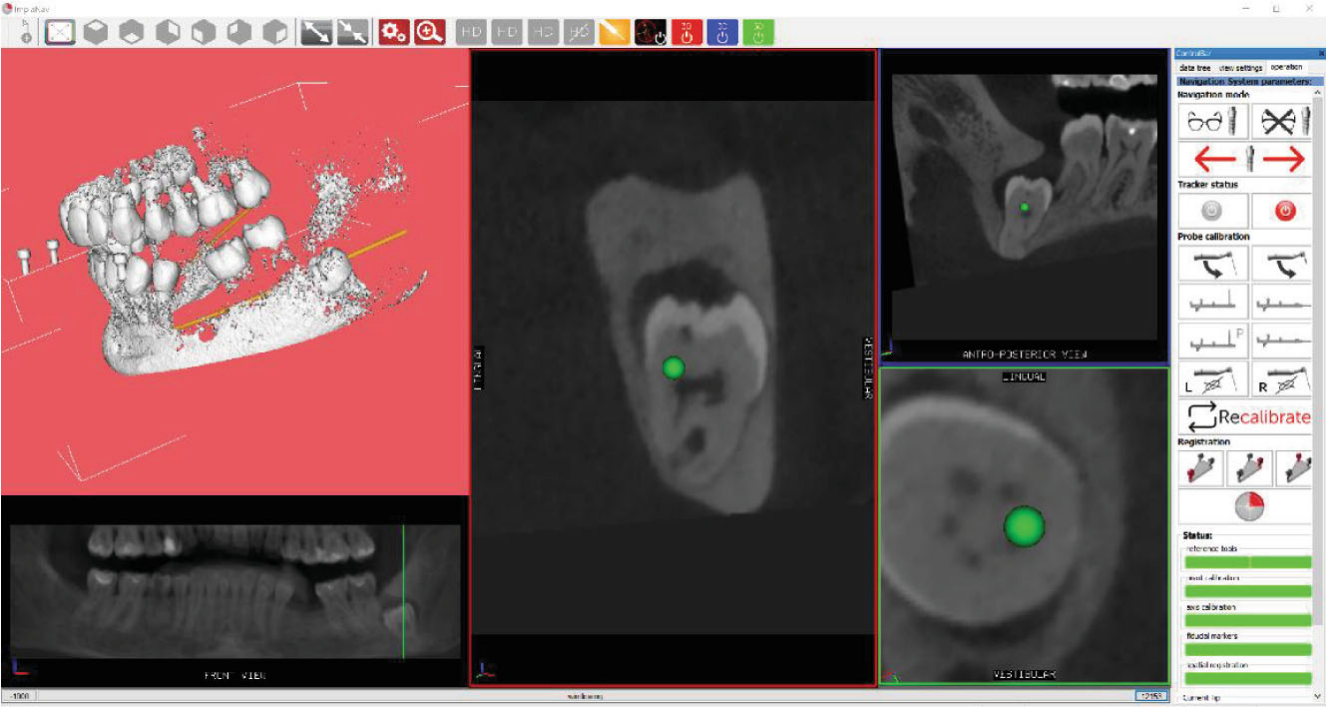
Intraoperative real-time images of the position of the surgical handpiece related to anatomical structures.

The preoperative CBCT was acquired with an intraoral reference device—a marker plate (MP) carrying radiopaque fiducials—seated in the mouth. Similar to an impression tray, the MP was temporarily stabilized to the dentition using a high-density elastomeric impression material (Ramitec; 3M ESPE, United States). Three sound teeth were included to ensure stable retention, and the anchorage site was selected after simulating any potential collision between the MP and the surgical handpiece trajectory. After imaging, the reference assembly was removed and stored, then reattached intraoperatively. The DICOM dataset was imported into the navigation software to delineate dental and mandibular anatomy, trace the mandibular canal and crown morphology, and enable real-time tracking of the bur during the intervention. On the day of surgery, after administering an inferior alveolar nerve block, the MP was reseated and, this time, the patient reference tool (RTp) remained attached. The RTp comprises retroreflective spheres arranged in a fixed geometry that can be detected by an infrared optical tracking camera (NDI, Waterloo, Canada) and recognized in real time from the 2D video frames. A second reference (RTh) was mounted via an adjustable ball-joint to both the contra-angle handpiece and the ultrasonic handpiece. The ball-joint allowed flexible positioning of the camera for an ergonomic, uninterrupted line of sight to the operative field and the display. For registration and calibration, the operator first held the instrument-mounted RTh within the camera's view for at least 3 s so it could be paired with the RTp. Subsequently, the fiducial markers on the MP were contacted sequentially using a dedicated calibration stylus/bur, enabling the software to register the tracked tool tip and axis to the CBCT-derived three-dimensional patient dataset (Figure 2).

Detailed descriptions of these procedures, as well as the surgical protocol, were reported in a previous publication presenting the 2-year follow-up of this case [25].

The coronectomy procedure was performed following the standardized protocol proposed by Monaco et al. [14], which includes a trapezoidal mucoperiosteal flap, removal of the crown, reduction of the roots to 3 mm below the alveolar crest, and primary tension-free wound closure.

After surgery, nonsteroidal analgesic medication was prescribed, to be taken only in case of pain and swelling. The patient was instructed to adhere to a soft diet for a few days, to maintain appropriate oral hygiene, and to rinse daily with a 0.2% chlorhexidine mouth wash.

**Figure 3:**
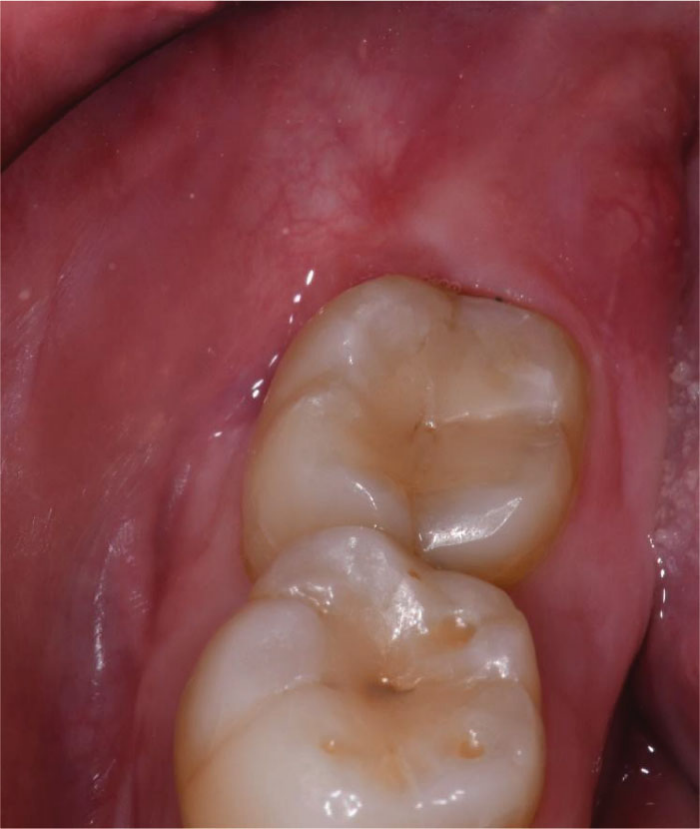
Clinical situation at the 5-year follow-up visit.

**Figure 4:**
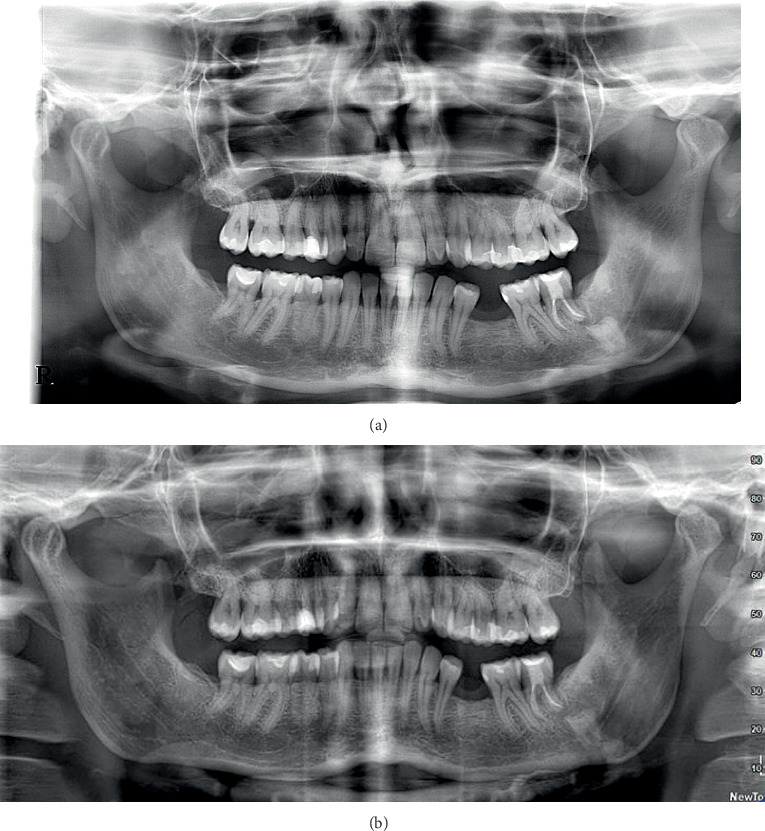
(a) Orthopantomography radiographic 2 years after coronectomy. (b) Orthopantomography radiographic 5 years after coronectomy.

At the 5-year follow-up, the surgical site exhibited complete mucosal healing with no signs of infection. No bleeding on probing was observed, and probing depths at the distal aspect of the second molar measured 3 mm buccally, 3 mm mesially, and 3 mm distally (Figure 3). A panoramic radiograph was obtained to assess the long-term outcome (Figure 4), showing that the residual roots of the third molar remained in situ without any signs of pathology. Additionally, new bone formation distal to the second molar and around the residual roots was evident.

## 3. Discussion

DN represents a novel adjunct in coronectomy, with only a handful of cases and pilot studies reported in the literature. This emerging technique leverages real-time guided instrumentation to enhance surgical precision and safety. In a pilot study by Zhang et al. [24], DN-enabled coronectomy achieved a root mean square deviation of only ~0.7 mm between the planned resection plane and the actual cut, indicating high precision. The authors concluded that DN improved the accuracy of coronectomy and minimized collateral tissue damage during third molar removal. Such precision is critical in high-risk mandibular third molars—even millimeter-scale accuracy can help avoid unintended contact with the inferior alveolar nerve or excessive bone removal. Our case corroborates these findings, as DN guidance allowed exact sectioning of the crown with no trauma to surrounding bone, contributing to uneventful healing.

Early clinical data suggest that DN-assisted third molar surgeries can reduce complication rates compared to conventional techniques. In a randomized trial on deeply impacted molars, Fangfang et al. [23] reported *no* nerve injuries or adjacent tooth damage in the DN group, versus several such injuries in the freehand group. The navigation-guided extractions also had significantly shorter operative times (22 vs. 36 min) despite a brief planning phase. These outcomes highlight the safety advantages of real-time guidance. Similarly, our DN-assisted coronectomy case exhibited zero complications over 5 years: no neuropathy, infection, or need for reintervention. This aligns with the low neuro-injury rates of standard coronectomy (inferior alveolar nerve injury ≈ 0.6%) [2], while suggesting that DN does not introduce new risks. Notably, conventional coronectomy literature reports that roughly 5% of cases may eventually require root removal (often due to migration or infection) [2]. In contrast, our case showed *no* root migration over 5 years, avoiding any secondary intervention. The absence of root migration—whereas minor upward movement (2–3 mm in the first year) is common in coronectomy [26]—may indicate that the precisely planned sectioning preserved the vitality and stability of the retained root. With respect to long-term outcomes, coronectomy may be followed by limited root migration (typically occurring within the first 6–24 months and then stabilizing), occasional late infections, or the need for secondary removal of the retained roots; pooled estimates suggest reoperation in approximately 3%–5% of cases, often linked to residual enamel or symptomatic migration.

Another important consideration is the learning curve and usability of DN in oral surgery. Adopting DN technology requires additional training and setup compared to routine practice. Tang et al. [27] emphasized that DN entails substantial upfront costs, comprehensive training, and extrapreoperative preparation time. Likewise, Emery et al. [28] noted an initial learning curve as a potential drawback to in-office navigation, along with the need for preoperative imaging and calibration. However, these initial investments may be offset by DN's role as a teaching tool—guiding less experienced surgeons through complex cases with enhanced visualization. Indeed, navigation allows the surgeon to “see” critical anatomy in real time, which can flatten the learning curve for difficult procedures. Our experience supports this; once the team became familiar with the system's workflow (planning, calibration, and guidance), the coronectomy was completed efficiently and with confidence in avoiding nerve injury. The system used in our case (ImplaNav, BresMedical) allowed for fiducial registration via a miniscrew inserted in the premaxilla, avoiding invasive anchorage in the mandible and simplifying surgical access. Over time, improvements in DN software and ergonomics are likely to streamline the process, further reducing setup time and making the technology more user-friendly in everyday practice. One additional consideration is that navigation-assisted surgery may alter operative ergonomics, requiring the surgeon to adapt to indirect visualization and potentially limiting intraoperative eye contact with the patient or assistants. These changes may initially disrupt the surgeon's workflow, although experience tends to mitigate such issues.

Finally, patient-reported outcomes with DN-assisted coronectomy appear favorable. Precise, minimally invasive execution can translate to reduced postoperative pain and faster recovery. Pellegrino et al. [17] reported that patients undergoing flapless, bone-preserving third molar extractions with DN required no postoperative analgesics, attesting to markedly low pain levels. In our case, the patient's recovery was similarly uneventful: pain was well controlled, and normal function was maintained during follow-up. Absence of dry socket or infection, combined with the preservation of bone and avoidance of a large surgical flap, likely contributed to rapid healing and high patient satisfaction. Although formal patient satisfaction surveys were not conducted, the 5-year trouble-free outcome—with the patient remaining symptom free and the tooth fragment integrally embedded in new bone—speaks to the success of the approach. Such outcomes compare favorably with conventional coronectomy, where postoperative pain is reported in about 20% of cases and occasional delayed root extractions can cause additional distress [2]. The DN-assisted technique thus not only prioritizes safety and precision but also may improve the overall patient experience by minimizing operative trauma [29].

In summary, while DN offers promising advantages in terms of surgical accuracy and real-time anatomical control, current evidence on its application in coronectomy remains limited. Most available data are derived from pilot studies or isolated case reports, including the present one, which—despite a favorable 5-year outcome—cannot provide generalizable conclusions. The additional costs, technical complexity, and learning curve associated with DN must also be carefully weighed.

## 4. Conclusion

DN technology has proven to be a valuable tool for managing complex third molars, particularly in cases where there is a high risk of IAN injury. The use of DN in coronectomy allows for precise control of surgical tools in relation to the radiological anatomy, thereby minimizing the risk of postoperative complications. In the long term, expected trajectories after coronectomy include stabilization of any early root migration, low rates of late infection, and generally favorable periodontal conditions distal to the second molar when protocols are respected; our 5-year outcome was consistent with this pattern. Given the single-case design, system-specific workflow, and imaging constraints at 5 years, broader conclusions are not possible; confirmatory prospective studies with standardized accuracy reporting and long-term imaging are warranted.

## Data Availability

The data used to support the findings of this study are available from the corresponding author upon request.
